# Impact of gender-biased parental perceptions on under-immunization in Eastern Sudan: a cross-sectional study

**DOI:** 10.3389/fgwh.2024.1337553

**Published:** 2024-10-29

**Authors:** Malaz Sulieman Abdallah, Taqwa Jumma, Yasir Ahmed Mohammed Elhadi, Majdi M. Sabahelzain

**Affiliations:** ^1^ Public Health Department, School of Health Sciences, Ahfad University for Women, Omdurman, Sudan; ^2^ Global Health Focus, Khartoum, Sudan; ^3^ Sydney School of Public Health, Faculty of Medicine and Health, University of Sydney, Sydney, NSW, Australia

**Keywords:** gender norms, vaccination, under-immunization, Kassala, Sudan

## Abstract

**Background:**

Despite global efforts, inequities in vaccine uptake remain, influenced by socioeconomic, geographic, cultural, and gender-related factors. In Eastern Sudan, gender disparities are acknowledged, particularly in livelihoods, but their impact on vaccination uptake is unclear. This study aimed to assess the effect of gender-biased parental perceptions on under-immunization among children in Kassala, Eastern Sudan.

**Methods:**

This study was a community-based cross-sectional in rural and urban districts of Kassala locality in Kassala State, Eastern Sudan in November 2022. Data were collected from parents using a pre-tested, structured questionnaire. The Chi-square or Fisher's exact test was conducted to assess the factors associated with under-immunization among children.

**Results:**

Data were collected from 400 parents. Our data reveal that most children were fully vaccinated with the three doses of the pentavalent vaccine (83%), while 14% were partially vaccinated. Findings showed that about one in five parents perceived male vaccination as more important than female vaccination. This parental perception of gender-based importance in vaccination was significantly associated with under-immunization among children (*p*-value = 0.049). Additionally, males in our study are fully vaccinated 5% more often than females. Socio-economic factors, including mothers’ education and households’ income level, were also significantly associated with the vaccination status of the children.

**Conclusion:**

This study shed light on the effect of gender norms and related determinants on equitable access to vaccinations for boys and girls alike. More research is needed to gain a better understanding of the gender norms related to vaccination and their long-term impact on immunization demand and resilience in this region.

## 1 Introduction

Despite global immunization efforts, inequities in vaccine uptake remain, influenced by socioeconomic, geographic, cultural, and gender-related factors, affecting equitable access to these crucial health services (1–3). The Immunization Agenda 2030 highlights the importance of comprehensive vaccine coverage for individual health and community disease resilience (4).

The WHO launched the Expanded Program on Immunization (EPI) in 1974 to provide vaccinations to children globally (5). However, vaccine accessibility remains problematic in Sub-Saharan Africa, where a significant number of children lack essential vaccinations, leading to high mortality rates from vaccine-preventable diseases (VPDs) (6, 7). Sudan's EPI, initiated in 1976, mandates vaccination for eligible groups, offering free immunizations to residents, including refugees (8, 9). Despite this, persistent conflicts, political upheaval, and socioeconomic disparities present considerable barriers to achieving vaccination goals in Sudan (10, 11).

Gender-based disparity in childhood immunizations is a public health challenge in some countries, with societal biases often resulting in a preference for vaccinating boys over girls (12–14). Such stereotypes, coupled with limited decision-making power for women, hinder access to vaccinations, particularly for female children, thereby increasing their vulnerability to VPDs (15–17).

A comprehensive analysis of childhood immunization in 52 countries revealed a connection between higher maternal empowerment and increased vaccination rates (18). A systematic review of factors influencing vaccination status in children (19) indicated that barriers related to gender include the cost of vaccinations, limited decision-making power for women, and time constraints due to maternal and social responsibilities. Moreover, restricted mobility, social norms, and factors related to the demand for vaccination, such as education, health literacy, and non-Western medical beliefs, also contribute to hindered access to vaccinations.

Despite the rollout of multiple vaccines in Sudan, vaccine-preventable illnesses continue to be a leading cause of mortality in children below five years of age in recent periods (20–22). In 2022, a national report indicated that the coverage for the first and the third doses of the pentavalent vaccine was 94% and 84%, respectively (23). Yet, a more detailed Simple Spatial Survey (S3M) published in 2020, which shows data specific to localities and states, revealed a lower vaccination rate, with just three-quarters of children (76%) receiving the first dose of the pentavalent vaccine (24). This survey also highlighted that Kassala state in Eastern Sudan had a significant proportion of under-immunized children (40%), in contrast, 23% of under-immunized children in Khartoum state.

Gender disparities are acknowledged in Eastern Sudan, particularly in livelihoods, but their impact on vaccination uptake is unclear. This study aimed to assess gender-biased parental perceptions’ influence on under-immunization among children in Kassala, Eastern Sudan. We used the third dose of the DTP-containing vaccine as a measure of under-immunization. It contains diphtheria, tetanus, pertussis, hepatitis B, and Haemophilus influenzae type b (Hib) vaccine. For programmatic purposes, Gavi uses DTP as a proxy for un-vaccination and under-immunization. Children need to receive three doses of the DTP vaccine to be considered fully immunized. DTP is administered through routine national immunization programs rather than campaigns (25). These findings can inform social and behavioral strategies to increase vaccine uptake in Eastern Sudan.

## 2 Methods

### 2.1 Study design and setting

This was a community-based cross-sectional study and conducted in rural and urban districts of Kassala locality in Kassala State, Eastern Sudan in November 2022. The State covers an area of 42,282 km^2^ with a population of approximately 2.5 million inhabitants. It is located in the eastern part of Sudan, bordered by the states of Eritrea and Ethiopia. It is subdivided into 11 localities. Most populations practice grazing in addition to agriculture.

### 2.2 Study population and sampling

The study population includes parents of at least one child aged (6–35 months). Either mothers or fathers were eligible for participation. Parents were asked to report about only one child to reduce the risk of recall bias. If there was more than one child in the same age range in the family, the parents/guardians were asked to report about only the youngest one. If both mother and father were available, they were asked to nominate one of themselves to complete the questionnaire.

To ensure that people from various socio-cultural and socioeconomic backgrounds (i.e., education and wealth level) are included in this study, we collected data from parents/guardians in two different rural and urban districts in Kassala; Tajouj village, and Alkhatmia district. Parents were selected in each district using consecutive sampling (convenience sample), as every parent meeting the criteria of inclusion (i.e., had a child in the age range) was included in the study until the required sample size was achieved from each district. The minimum sample size was estimated at 384. To cover for possible drop-out due to refusal to answer some crucial questions, a total of 400 parents were selected for the study (i.e., 200 per district). The sample size was computed using the following formula:

$$\left[n=\frac{z^{2}\mathrm{pq}}{d^{2}}\;\right]$$

Where, n = sample size. z = (1 - *α*), is the z-score corresponding to a 95% confidence interval and was computed as 1.96. p = Expected probability of pentavalent vaccination coverage in the rural areas in Kassala, which is unknown, therefore it was estimated at 50%. q = (1- p) or 50%. d = desired margin of error or 0.05.

### 2.3 Data collection

Data were collected using the Behavioral and Social Drivers of Vaccination (BeSD) survey (26), which was translated to Arabic, and adapted and pre-tested in the local context. This survey, developed by the WHO, measures four underlying factors: thoughts and feelings, social processes, motivation, and practical issues. The question about perception toward gender-biased vaccination was adapted from a question about decision-making autonomy for vaccination in the (BeSD) survey. The questionnaire was interviewer-administered. The questionnaire for the study included parent's socio-demographic characteristics, child characteristics, mother's educational level, household income (was measured as self-ranking), and perception towards gender-biased vaccination, which were used as independent variables. The uptake of the pentavalent vaccine was used as the dependent variable, which was measured as fully immunized (being vaccinated with three doses), partially immunized (being vaccinated with one or two doses) and not immunized who have not received any dose of pentavalent.

### 2.4 Data analysis

Data analysis was performed using Statistical Package for Social Sciences (SPSS) software (Version 26). The Chi-square or Fisher's exact (when the count is <5 in a cell) test was conducted to assess the factors associated with under-immunization among children. We focused on under-immunization rather than non-vaccination because we found that the proportion of under-immunized children is 14%, which is much higher than the proportion of unvaccinated children (2%). We excluded from the Chi-square test those who were not vaccinated, didn't know their vaccination status and missing data (e.g., Mother's education). A *p*-value of <0.05 is considered statistically significant.

### 2.5 Ethical consideration

Ethical approval was obtained from the Ahfad University for Women Ethical Committee (IRB). Permission to enter the communities was obtained from the community leaders in the rural districts. Written and/or verbal informed consent was obtained from each participant.

## 3 Results

### 3.1 Socio-demographic characteristics of the parents and children

As shown in Table 1, a total of 400 parents of children aged 6–35 months were interviewed with 100% response rate. Half of them (50.0%) are from the urban district. The majority of the participants (91.8%) were mothers, and (92.5%) of them were not employed. The mean age of mothers was 27.6 (SD = 5.36). We surveyed the parents about the level of education of the mothers; results revealed that over half of the mothers (54.7%) had attended primary school, while 19.5% had never attended school. Concerning marital status, the vast majority (95.2%) of the parents are currently married. Half of the participants (50.2%) self-ranked their household income as medium. About half of the households (53.0%) have only one child under the age of five, followed by 38.3% of households having 2 children. Of the children whose vaccination status was recorded, 52.8% were male.

**Table 1:** Socio-demographic characteristics of the parents and children (*N* = 400).

		*N*	%
Residency	Urban	200	50.0%
	Rural	200	50.0%
Parent	Mother	367	91.8%
	Father	14	3.5%
	Others	19	4.8%
Mothers’ education	Not educated	77	19.3%
	Primary	217	54.3%
	Secondary	65	16.2%
	University	38	9.5%
	Missing/Not responded	3	0.7%
Mother employment	Not employed	370	92.5%
	Employed	30	7.5%
Marital status	Married	381	95.3%
	Widow	5	1.3%
	Divorced	10	2.5%
	Separated	4	1.0%
Household's income level (self-ranking)	High	6	1.5%
	Medium	201	50.2%
	Low	108	27.0%
	Very low	85	21.3%
Number of children	1	212	53.0%
	2	153	38.3%
	3	29	7.2%
	4 and more	6	1.5%
Sex of the child	Male	211	52.8%
	Female	189	47.3%

### 3.2 Vaccination status

**Figure 1:**
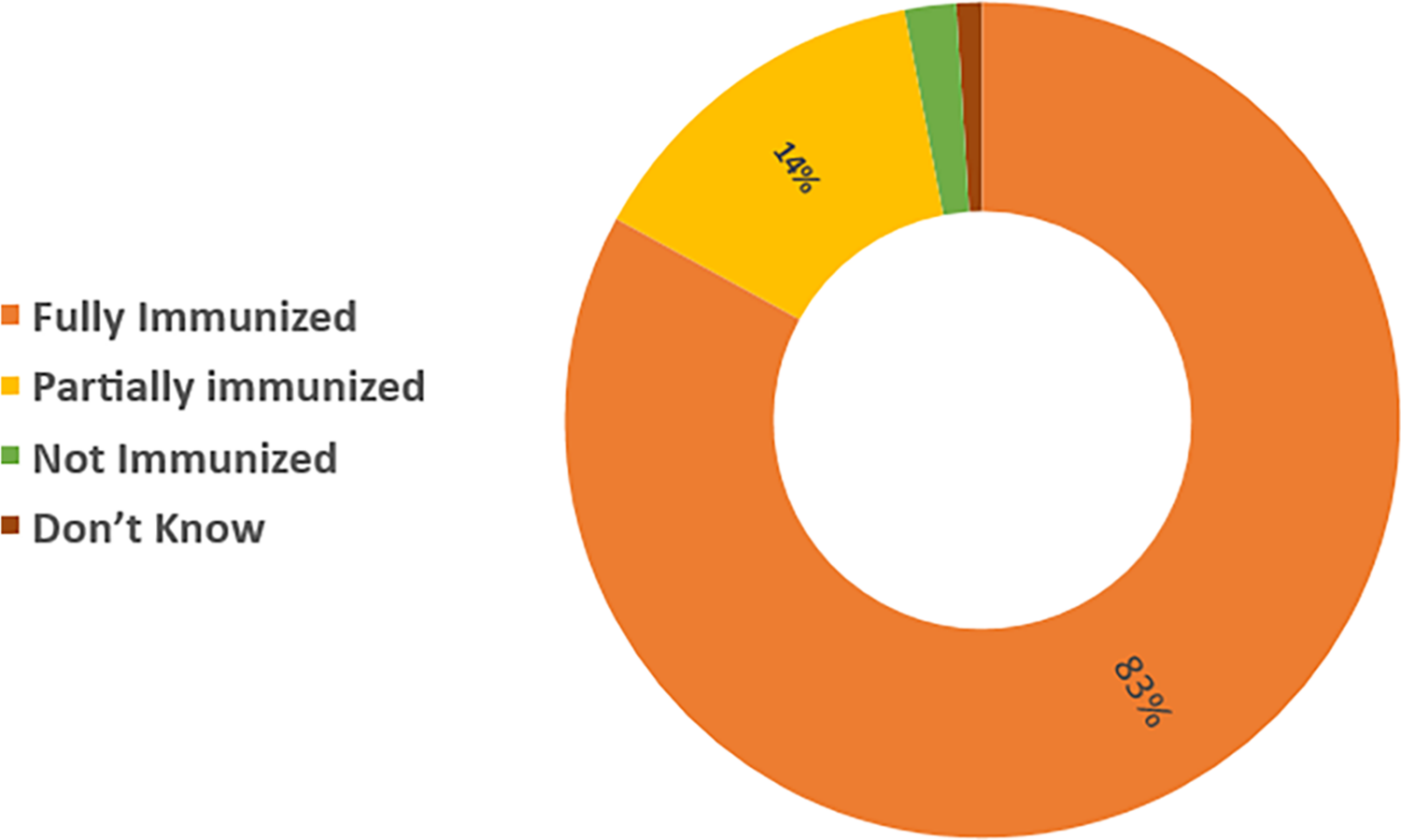
Vaccination status of the children in rural and urban Kassala, Sudan.

Figure 1 shows that the majority of the children (83%) were fully vaccinated with the pentavalent vaccine (i.e., 3 doses), while 14% were vaccinated with either one or two doses (Partially immunized) and 2% were not vaccinated.

### 3.3 Parental perception towards gender and vaccination

**Figure 2:**
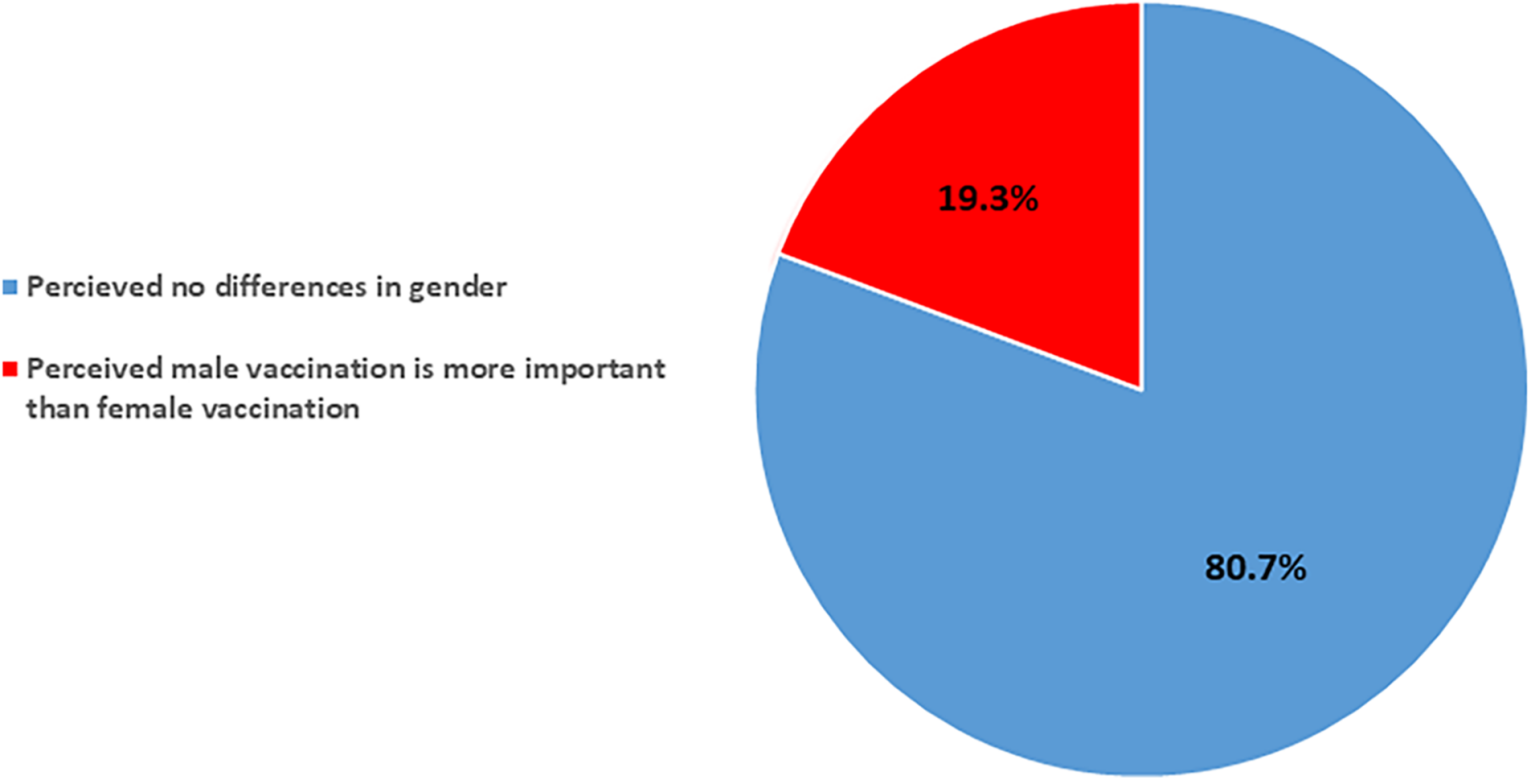
Parental perceptions of gender-based importance in vaccination in rural and urban Kassala, Sudan.

Figure 2 shows that about 1 in 5 participants (19.3%) perceived male vaccination as more important than female vaccination. We did further analysis to assess the association between perceiving male vaccination as more important than female vaccination and the socio-economic status of the parents, including their residence (i.e., rural or urban) and their income level, but there were no significant associations.

### 3.4 Factors associated with children's vaccination status

Table 2 shows that socioeconomic factors, including mothers’ education (*p* = 0.001) and household income level (*p* = 0.026) were significantly associated with children's vaccination status. Mothers with primary education were more likely to partially vaccinate their children with the pentavalent vaccine, followed by secondary, and non-educated mothers. Those who self-ranked their household's income as low or very low were more likely to have under-immunized children.

**Table 2:** Association of socioeconomic factors and parent perception with the immunization status of children in Kassala, Sudan (*N* = 389).

		Immunization status				*P*-value
		Partially immunized		Fully immunized		
		*N*	%	*N*	%	
Residency	Urban	27	13.8%	169	86.2%	0.725
	Rural	29	15.0%	164	85.0%	
Mothers’ education	Not educated	4	5.6%	68	94.4%	0.001*
	Primary	46	21.7%	166	78.3%	
	Secondary	5	7.7%	60	92.3%	
	University	1	2.7%	36	97.3%	
Household income level (self-ranking)	High	1	20.0%	4	80.0%	0.026*
	Medium	18	9.1%	180	90.9%	
	Low	21	20.2%	83	79.8%	
	Very low	16	19.5%	66	80.5%	
Number of children	1	35	16.8%	173	83.2%	0.268
	2	19	12.9%	128	87.1%	
	3	1	3.6%	27	96.4%	
	4 and more	1	16.7%	5	83.3%	
Sex of the child	Male	24	11.8%	179	88.2%	0.131
	Female	32	17.2%	154	82.8%	
Parental perceptions of gender-based importance in vaccination	Perceived male vaccination is more important than female vaccination	16	21.6%	58	78.4%	0.049*
	Perceived no difference in gender	40	12.7%	275	87.3%	

Furthermore, the parental perception of the importance of male vaccination showed a significant association with children's vaccination status (*p* < 0.049). Parents who perceived male vaccination as more important than female vaccination are more likely to partially vaccinate their children.

There were no significant associations between vaccination status and the residence of the household, the number of children, or the sex of the child whose vaccination status was recorded.

## 4 Discussions

This paper explores the gender-specific determinants that impact the uptake of the pentavalent vaccine in rural and urban areas in Kassala State, Sudan.

The pentavalent vaccine includes diphtheria, tetanus, and pertussis (DTP), as well as hepatitis B and *H. Influenzae* type b (Hib). Children receive three doses of DTP as a trivalent or pentavalent vaccine within the first months of life. During vaccination sessions, oral polio vaccine (OPV) doses are administered along with DTP. Gavi linked zero-dose and under-immunization definitions with DTP vaccination for operational purposes, and it is used as a proxy for all routine childhood vaccinations (25).

Our data reveal that most children were fully vaccinated with three doses of the pentavalent vaccine (83%), while 14% were partially vaccinated, receiving one or two doses. Only 2% had received no pentavalent doses. The under-immunization in our study is lower than the state-level figure (60%) (16). This may be attributed to the area of study, which is commonly known as the capital of the Kassala state, where a relatively sufficient number of health services can be accessed.

Alarmingly, our findings showed that about one in five parents perceived male vaccination as more important than female vaccination. This parental perception of gender-based importance in vaccination was significantly associated with under-immunization among children (*p*-value = 0.049). Further, males in our study are fully vaccinated 5% more often than females. This preference for male vaccination is likely rooted in societal and cultural gender norms in the Eastern Sudan communities. Additionally, these findings are a stark indicator of gender inequality in healthcare decision-making and reflect disparities in the uptake of immunization services in Sudan. This bias was significantly related to the children's vaccination status, suggesting a gender-based discrepancy in vaccination coverage that disadvantages female children. Such disparities highlight the urgent need for interventions to eliminate gender biases from health services, ensuring equitable health outcomes for all children (4, 20, 22, 27, 28).

Furthermore, the study underscores a mother's education as a pivotal factor in her child's likelihood of being vaccinated. This suggests that maternal literacy impacts health decisions, which can have gender-specific effects depending on societal norms (29, 30). Evidence shows that higher maternal empowerment is linked to increased vaccination rates (18), while limited decision-making power for women was identified as one of the gender-related barriers that influences children's vaccination status (19).

Educational initiatives directed at mothers, especially those with limited schooling, are essential not only to improving the overall childhood vaccination rate but also to bridging the knowledge gap and promoting equal vaccination opportunities for both boys and girls (31).

Household income was also identified as a significant determinant of vaccination status. However, we did not find a significant association between income level and perceiving male vaccination as more important than female vaccination. In contrast, another study suggests that economic disparities may intersect with gender disparities, as lower-income families might differentially allocate healthcare resources between sons and daughters (32). Tackling this issue requires strategies that make vaccinations accessible to all economic segments, which may help improve the vaccination rate.

### 4.1 Limitation

We acknowledge some limitations related to our study; therefore, the study's findings should be interpreted within the context of this study. One of the limitations of this study is that there was unintentional selection bias due to consecutive sampling to recruit participants until the pre-determined sample size was reached from both districts. Additionally, from a gender perspective, most of the participants were female; this may be attributed to the time when the interviews were conducted (i.e., men at work). It was observed that fathers’ perceptions, perspectives, and decision-making roles regarding vaccination were missing due to their absence during the study. However, in Sudanese culture, like many other African countries, mothers are primarily responsible for the health and well-being of their children and family and, hence, have a better understanding of the health situation (33). Therefore, some parents preferred that the study should include mothers rather than fathers. We suggested future research to explore the perspectives of a broader range of parents, including fathers.

Given the study's purpose of shedding light on the role of gender norms on vaccination and its possible impact on children's vaccination, we performed only the chi-square test to examine the relationship between independent variables and the dependent variable. However, we did not go further in doing a logistic regression, which could be helpful in analyzing categorical variables and better understanding the predictors of immunization status.

## 5 Conclusion

This study emphasizes the importance of confronting gender biases in healthcare to guarantee equitable access to vaccinations for boys and girls alike. Gender norms related to vaccination in Sudan are not well-documented. However, data collectors anecdotally reported that some participants believe that male children should be vaccinated to protect them, as they tend to spend more time outside the house. More qualitative research is needed to gain a better understanding of the gender norms related to vaccination and their long-term impact on immunization demand and resilience in this region.

## Data Availability

The raw data supporting the conclusions of this article will be made available by the authors, without undue reservation.
