# Supplementary material for: Impact of gender-biased parental perceptions on under-immunization in Eastern Sudan: a cross-sectional study
Source: Front Glob Womens Health. 2024 Oct 29;5:1337553. doi: 10.3389/fgwh.2024.1337553 (PMC11554609; doi:10.3389/fgwh.2024.1337553)
Supplement: Supplementary file 1 [file Table1.docx]

*جامعة الأحفاد للبنات*

*مدرسة العلوم الصحية*

*كلية الصحة العامة*

**إستبيان تقييم العوامل الاجتماعية والسلوكية للتطعيم الاطفال في المناطق الريفي**

**ة والحضرية في ولاية كسلا نوفمبر 2022**

| **البيانات السكانية والأجتماعية للأسرة** | | | |
| --- | --- | --- | --- |
| HH0 | المناطق الريفية او الحضرية في كسلا | | 1- مناطق حضرية |
|  |  |  | 2- مناطق ريفية |
| HH1 | من الذى أجاب على الإستبيان؟ | | 1- الأم |
|  |  |  | 2- الاب |
|  |  |  | 3- أخرى، حدد (................................................) |
| HH2 | عدد أفراد الأسرة؟ | | ............. |
| HH3 | عدد الأطفال دون سن الخامسة؟ | | ............. |
| HH4 | من هو رب المنزل (عائل الأسرة) | | 1- امراْة |
|  |  |  | 2-رجل |
| HH5 | كم عمر الأم؟ | | .............. |
| HH6 | كم عمر الأب؟ | | .............. |
| HH7 | ما هي قبيلة الأب؟ | | ............. |
| HH8 | ما هي قبيلة الأم | | ............. |
| HH9 | ماهو مستوى تعليم الأم؟ | | 1- غير متعلم |
|  |  |  | 2-خلوة |
|  |  |  | 3- أساس |
|  |  |  | 4- ثانوى |
|  |  |  | 5- جامعى |
|  |  |  | 6- ماجستير/ دكتوراة |
|  |  |  | 7- لا أعرف |
| HH10 | ماهو مستوى تعليم الأب؟ | | 1-غير متعلم |
|  |  |  | 2- خلوة |
|  |  |  | 3- أساس |
|  |  |  | 4- ثانوى |
|  |  |  | 5- جامعى |
|  |  |  | 6-ماجستير/ دكتوراة |
|  |  |  | 7- لا أعرف |
| HH11 | الحالة الإجتماعية | | 1- متزوجة/ متزوج |
|  |  |  | 2-أرملة |
|  |  |  | 3-مطلقة |
|  |  |  | 4-منفصلين/ مهجورة |
| HH12 | مهنة الأم؟ | | 1- ربة منزل |
|  |  |  | 2- طالبة |
|  |  |  | 3- عاملة |
|  |  |  | 4- موظفة |
|  |  |  | 5-متخصصة (مهندس، طبيب، محامى..) |
|  |  |  | 6-عمل حر ( يمتلك عمله الخاص) |
|  |  |  | 7-أخرى (........................................) |
|  |  |  | 8- لا أعرف |
| HH13 | مهنة الأب؟ | | 1-عاطل عن العمل |
|  |  |  | 2-طالب |
|  |  |  | 3/-عامل |
|  |  |  | 4- موظف |
|  |  |  | 5-متخصص (مهندس، طبيب، محامى..) |
|  |  |  | 6-عمل حر ( يمتلك عمله الخاص) |
|  |  |  | 7- أخرى (........................................) |
|  |  |  | 8-لا أعرف |
| HH14 | **فى اعتقادك ماهو مستوى دخل أسرتك**؟ | | 1-عالى جدا |
|  |  |  | 2عالى |
|  |  |  | 3- وسط |
|  |  |  | 4-ضعيف |
|  |  |  | 5-ضعيف جدا |
| HH15 | ماهو عمر الطفل المراد التحدث عنه **(يجب أن يكون عمره بين 12شهر و23 شهر)** | | .................... |
| HH16 | جنس الطفل؟ | | 1-ولد |
|  |  |  | 2- بنت |
| HH17 | ترتيب الطفل بين أخوانه | | ………………. |
| **أسئلة عن التطعيم** | | | |
| Q1 | هل لديك كرت تطعيم لطفلك مكتوب فيه أسم الطفل (الذى عمره 12-23 شهر) | نعم (تمت رؤية الكرت) | |
|  |  | نعم (لم يتم رؤية الكرت) | |
|  |  | لا يوجد كرت | |
|  |  | لا أعرف | |
| Q2 | هل الطفل تم تطعيمه (تحصينه) بلقاح الخماسي, (DPT)  (من الكرت أو من احد الوالدين) | 1-لم يتم تطعيمه (اذهب الى السؤال رقم 3) | |
|  |  | 2- مطعم جزئيا :  -جرعة واحدة فقط  -جرعتين | |
|  |  | 3-تطعيم كامل (ثلاثة جرعات) | |
|  |  | 4-لا اعرف (ليس متاكدا) | |
| Q3 | لماذا قررت أن لا تطعم/ تحصن طفلك بلقاح الحصبة | …………………………………………………………………  ………………………………………………………………… | |
| Q4 | هل تعتقد أن تطعيم الذكورأهم من تطعيم الاناث؟ | 1-نعم | |
|  |  | 2-لا | |
| Q5 | لدى السودان جدول للقاحات الموصي بها للأطفال ,هل تريد أن يحصل طفلك على كل اللقاحات أو بعضها ام لا شيء منها؟ | 1- الكل | |
|  |  | 2-البعض | |
|  |  | 3- لا شي | |
| Q6 | في إعتقادك ما مدى اهمية اللقاحات لطفلك؟ | 1-ليست مهمة على الاطلاق | |
|  |  | 2-مهمة قليلا | |
|  |  | 3- متوسطة الاهمية | |
|  |  | 4- مهمة جدا | |
| Q7 | الي أي مدى تعتقد أن التطعيم امن لطفلك؟ | 1-ليست أمنة علي الاطلاق | |
|  |  | 2/ امنة قليلا | |
|  |  | 3-متوسطة الأمان | |
|  |  | 4- أمنة جدا | |
| Q8 | الي أي مدى تثق بموظفي الصحة الذين يعطون طفلك اللقاح؟هل تقول أن ثقتك........ | 1-لاأثق | |
|  |  | 2- تقتك قليلة | |
|  |  | 3-ثقتك متوسطة | |
|  |  | 4-تثق جدا | |
| Q9 | هل تعتقد أن معظم الأباء الذين تعرفهم يقومون بتطعيم أطفالهم؟ | 1-نعم | |
|  |  | 2-لا | |
| Q10 | هل تعتقد أن معظم أفراد عائلتك وأصدقائك المقربين يؤيدون تطعيم طفلك ؟ | 1-نعم | |
|  |  | 2- لا | |
| Q11 | هل تعتقد أن قادتك الدينيين يؤيدون تطعيم طفلك؟ | 1-نعم | |
|  |  | 3-لا | |
| Q12 | هل تعتقد أن قادة مجتمعك يؤيدون تطعيم طفلك؟ | 1-نعم | |
|  |  | 2-لا | |
| Q13 | هل يوصي العاملون الصحييون بتطعيم طفلك؟ | 1-نعم | |
|  |  | 2-لا | |
| Q14 | هل سبق وأن تم الإتصال بك بشأن موعد تلقي طفلك التطعيم؟ | 1-نعم | |
|  |  | 4- لا | |
| Q15 | إذا حان موعد تطعيم طفلك فهل تحتاج الأم الي إذن لأخذ طفلك الي المركز الصحي؟ | 1-نعم | |
|  |  | 2-لا | |
| Q16 | هل تعلم الى اين تذهب للحصول على تطعيم لطفلك؟ | 1-نعم | |
|  |  | 2-لا | |
| Q17 | - إذا كانت الإجابة نعم اين يتم التطعيم ؟ | ......................... | |
| Q18 | هل سبق وأن أخذت طفلك للتطعيم شخصيا؟ | 1-نعم | |
|  |  | 2-لا | |
| Q19 | هل سبق وأن تم إرجاعك عندما أخذت طفلك للتطعيم؟ | 1-نعم | |
|  |  | 2-لا | |
| Q20 | ما مدى سهولة حصول طفلك علي خدمة التطعيم؟ | 1- ليس سهل علي الإطلاق | |
|  |  | 2-سهل قليلا | |
|  |  | 3-متوسط السهولة | |
|  |  | 4-سهل جدا | |
| Q21 | ما مدى سهولة دفع ثمن التطعيم؟ ( الثمن يتضمن أي مدفوعات المركز الصحي , تكلفة الوصول لمكان  التطعيم ,وتكلفة أخذ الوقت بعيدا عن العمل )؟ | 1-ليس سهل علي الإطلاق | |
|  |  | 2-سهل قليلا | |
|  |  | 3-متوسط السهولة | |
|  |  | 4-سهل جدا | |
| Q22 | ما الذي يصعب حصولك علي خدمة التطعيم لطفلك؟ | 1- لا شي ليس صعب | |
|  |  | 2-يصعب الذهاب الي العيادة | |
|  |  | 3- وقت فتح العيادة غير مريح | |
|  |  | 4-المركز الصحي في بعض الاحيان ترجع الأشخاص من غير تطعيم | |
|  |  | 5-زمن الإنتظار في المركز الصحي يأخذ وقت طويل | |
|  |  | 6-أو يوجد شيء أخر الإجابة................................. | |
| Q23 | الي اي مدي انت راض عن خدمة التطعيم في منطقتك؟ | 1-غير راض | |
|  |  | 2-راض قليلا | |
|  |  | 3-متوسط الرضي | |
|  |  | 4-راض جدا | |
