# Supplementary material for: Impact of gender-biased parental perceptions on under-immunization in Eastern Sudan: a cross-sectional study
Source: Front Glob Womens Health. 2024 Oct 29;5:1337553. doi: 10.3389/fgwh.2024.1337553 (PMC11554609; doi:10.3389/fgwh.2024.1337553)
Supplement: Supplementary file 2 [file Table2.docx]

**Ahfad University for Women**

**School of Health Sciences**

**Public Health Major**

**Questionnaire about Assessment of Social and Behavioral Factors of Zero-Dose and under Immunization among Children in Rural and Urban Area of Kassala, November 2022**

| **Socio-demographic data of the Household** | | |
| --- | --- | --- |
| **Code** | **Question** | **Option** |
| HH0 | The rural or Urban area of Kassala? | **………………..** |
| HH1 | Answers the questionnaire? | 1/ Mother |
|  |  | 2/ Father |
|  |  | 3/ Other, mention (…………................) |
| HH2 | Family number? | …………. |
| HH3 | The number of children under the age of five years? | …………... |
| HH4 | Who is the head of the family (the family breadwinner)? | 1/ Male |
|  |  | 2/ Female |
| HH5 | Age of the mother? | ………………………….. |
| HH6 | Age of the father? | …………………………. |
| HH7 | Mother`s ethnicity? | ……………………….. |
| HH8 | Father`s ethnicity? | ……………………….. |
| HH9 | Level of mother education? | 1/Uneducated/ illiterate |
|  |  | 2/Khalwa (Religious school) |
|  |  | 3/Primary school |
|  |  | 4/ Secondary school |
|  |  | 5/ University |
|  |  | 6/Post graduated |
|  |  | 7/ I don’t know |
| HH10 | Level of father education? | 1/ Uneducated/illiterate |
|  |  | 2/ Khalwa (Religious school) |
|  |  | 3/ Primary school |
|  |  | 4/ Secondary school |
|  |  | 5/ University |
|  |  | 6/Post graduated |
|  |  | 7/ I don’t know |
| HH11 | Social status | 1/ Married |
|  |  | 2/ Widow |
|  |  | 3/ Divorced |
|  |  | 4/ Separated |
| HH12 | Mother’s profession | 1/ Housewife |
|  |  | 2/ student |
|  |  | 3/ Worker |
|  |  | 4/Officer |
|  |  | 5/Professional (Engineer, Doctor, Lawyer…….) |
|  |  | 6/ Self-employed |
|  |  | 7/ Others (…………...) |
| HH13 | Father's Employment | 1/Unemployed |
|  |  | 2/ Student |
|  |  | 3/ Worker |
|  |  | 4/ Officer |
|  |  | 5/ Professional(Engineer ,Doctor, Lawyer) |
|  |  | 6/ Self-employed |
|  |  | 7/ Others, specify (……………) |
|  |  | 8/ I don’t know |
| HH14 | How do you rank your household’s income level | 1/ Very high |
|  |  | 2/ High |
|  |  | 3/ Medium |
|  |  | 4/ Law |
|  |  | 5/ Very law |
| HH15 | Age of the child of whom the respondent will answer the questionnaire (should be aged 12- 23 months (1-2 years): | ………………….. |
| HH16 | Sex of the child (aged 12- 23 months (1-2years) | 1/ Male |
|  |  | 2/ Female |
| HH17 | The rank of this child (aged 12- 23 months (1-2 years) | ………………. |

| **Vaccination** | | |
| --- | --- | --- |
| **Code** | **Question** | **Option** |
| Q1 | Do you have a card where (name of the youngest child)’s vaccinations are written down? | 1/Yes, seen |
|  |  | 2/ Yes, not seen |
|  |  | 3/ No card |
|  |  | 4/I don’t know |
| Q2 | Has the child been vaccinated with the Pentavalent vaccine(from the card or one of the parents) | 1/ Unvaccinated **(Skip to Q3)** |
|  |  | 2/Partially vaccinated (One dose, or two-dose) |
|  |  | 3/ Fully vaccinated (three doses) |
|  |  | 4/ I don't Know/ No response |
| Q3 | Why did you decide NOT to vaccinate your child with the Pentavalent vaccine?  **(This question is only for Parents/ Guardians who refused to vaccinate their child with the measles vaccine)** | ………………………………………  ………………………………………  ………………………………………  ……………………………………………………………………………… |
| Q4 | Do you think the male vaccine important than the female vaccine? | 1/Yes |
|  |  | 2/No |
| Q5 | Sudan has a schedule of recommended vaccines for children. Do you want your child to get none of these vaccines, some of these vaccines, or all of these vaccines? | 1/Non |
|  |  | 2/Some |
|  |  | 3/All |
| Q6 | How important do you think vaccines are for your child’s health? Would you say… | 1/Not at all important |
|  |  | 2/A little important, |
|  |  | 3/Moderately important |
|  |  | 4/Very important |
| Q7 | How safe do you think vaccines are for your child? Would you say… | 1/ Not at all safe |
|  |  | 2/ A little safe |
|  |  | 3/Moderately safe, or |
|  |  | 4/Very safe |
| Q8 | How much do you trust the health workers who give children vaccines? Would you say you trust them… | 1/Not at all |
|  |  | 2/A little |
|  |  | 3/Moderately |
|  |  | 4/Very much |
| Q9 | Do you think most parents you know get their children vaccinated? | 1/Yes |
|  |  | 2/No |
| Q10 | Do you think most of your close family and friends want you to get your child vaccinated? | 1/Yes |
|  |  | 2/No |
| Q11 | Do you think your religious leaders want you to get your child vaccinated? | 1/Yes |
|  |  | 2/No |
| Q12 | Do you think your community leaders want you to get your child vaccinated? | 1/Yes |
|  |  | 2/No |
| Q13 | Has a health worker recommended your child be vaccinated? | 1/N0 |
|  |  | 2/Yes |
| Q14 | Have you ever been contacted if your child is due for vaccination? | 1/No |
|  |  | 2/Yes |
| Q15 | If it was time for your child to get vaccinated, would the mother need permission to take your child to the clinic? | 1/No |
|  |  | 2/Yes |
| Q16 | Do you know where to go to get your child vaccinated? | 1/No |
|  |  | 2/Yes |
| Q17 | If the answer was yes, where is the vaccination done? | ………………………… |
| Q18 | Have you personally ever taken your child to get vaccinated? | 1/No |
|  |  | 2/Yes |
| Q19 | Have you ever been turned away when you tried to get your child vaccinated? | 1/No |
|  |  | 2/Yes |
| Q20 | How easy is it to get vaccination services for your child? Would you say …. | 1/Not at all easy, |
|  |  | 2/A little easy |
|  |  | 3/Moderately easy, or |
|  |  | 4/Very easy |
| Q21 | How easy is it to pay for vaccination? When you think about the cost, please consider any payments to the clinic, the cost of getting there, plus the cost of taking time away from work. Would you say…. | 1/Not at all easy, |
|  |  | 2/A little easy |
|  |  | 3/Moderately easy, or |
|  |  | 4/Very easy |
| Q22 | What makes it hard to get vaccination services for your child? Would you say…. | 1/Nothing, it’s not hard, |
|  |  | 2/Getting to the clinic is hard, |
|  |  | 3/The clinic opening times are inconvenient |
|  |  | 4/The clinic sometimes turns people away without vaccinating |
|  |  | 5/ The waiting time in the clinic takes too long |
|  |  | 6/ or Is there something else?  Answer…………………………… |
| Q23 | How satisfied are you with the vaccination services? Would you say…. | 1/Not at all satisfied, |
|  |  | 2/A little satisfied |
|  |  | 3/Moderately satisfied, or |
|  |  | 4/Very satisfied |
